# Implications of subclinical tuberculosis for vaccine trial design and global effect

**DOI:** 10.1016/S2666-5247(24)00127-7

**Published:** 2024-10

**Authors:** Gavin J Churchyard, Rein M G J Houben, Katherine Fielding, Andrew L Fiore-Gartland, Hanif Esmail, Alison D Grant, Molebogeng X Rangaka, Marcel Behr, Alberto L Garcia-Basteiro, Emily B Wong, Mark Hatherill, Vidya Mave, Alemnew F Dagnew, Alexander C Schmidt, Willem A Hanekom, Frank Cobelens, Richard G White

**Affiliations:** a Aurum Institute NPC, Houghton, Parktown, South Africa; b Department of Medicine, Vanderbilt University, Nashville, TN, USA; c School of Public Health, Faculty of Health Sciences, University of Witwatersrand, Johannesburg, South Africa; d Department of Infectious Disease Epidemiology, London School of Hygiene & Tropical Medicine, London, UK; e TB Modelling Group, TB Centre, London School of Hygiene & Tropical Medicine, London, UK; f TB Centre, London School of Hygiene & Tropical Medicine, London, UK; g Department of Clinical Research, London School of Hygiene & Tropical Medicine, London, UK; h University of Washington, Seattle, WA, USA; i MRC Clinical Trials Unit, University College London, London, UK; j Division of Infection and Immunity, University College London, London, UK; k WHO Collaborating Centre for TB Research and Innovation, Institute for Global Health, University College London, London, UK; l McGill International TB Centre, McGill University, Montreal, QC, Canada; m ISGlobal, Hospital Clínic - Universitat de Barcelona, Barcelona, Spain; n CIDRI-AFRICA, School of Public Health, Institute of Infectious Disease and Molecular Medicine, University of Cape Town, Cape Town, South Africa; o South African Tuberculosis Vaccine Initiative, Department of Pathology and Institute of Infectious Disease and Molecular Medicine, University of Cape Town, Cape Town, South Africa; p Centro de Investigação em Saúde de Manhiça (CISM), Maputo, Mozambique; q Africa Health Research Institute, KwaZulu-Natal, Durban, South Africa; r Centro de Investigación Biomédica en Red de Enfermedades Infecciosas (CIBERINFECT), Barcelona, Spain; s Division of Infectious Diseases, Heersink School of Medicine, University of Alabama at Birmingham, Birmingham, AL, USA; t Byramjee–Jeejeebhoy Government Medical College, Johns Hopkins University Clinical Research Site, Pune, India; u Gates Medical Research Institute, Cambridge, MA, USA; v Department of Global Health and Amsterdam Institute for Global Health and Development, Amsterdam University Medical Center, University of Amsterdam, Amsterdam, Netherlands

## Abstract

Tuberculosis is a leading cause of death from an infectious agent globally. Infectious subclinical tuberculosis accounts for almost half of all tuberculosis cases in national tuberculosis prevalence surveys, and possibly contributes to transmission and might be associated with morbidity. Modelling studies suggest that new tuberculosis vaccines could have substantial health and economic effects, partly based on the assumptions made regarding subclinical tuberculosis. Evaluating the efficacy of prevention of disease tuberculosis vaccines intended for preventing both clinical and subclinical tuberculosis is a priority. Incorporation of subclinical tuberculosis as a composite endpoint in tuberculosis vaccine trials can help to reduce the sample size and duration of follow-up and to evaluate the efficacy of tuberculosis vaccines in preventing clinical and subclinical tuberculosis. Several design options with various benefits, limitations, and ethical considerations are possible in this regard, which would allow for the generation of the evidence needed to estimate the positive global effects of tuberculosis vaccine trials, in addition to informing policy and vaccination strategies.

## Introduction

Tuberculosis remains a global health threat and a leading cause of death from an infectious agent.^1^ New tuberculosis vaccines are urgently needed to end the tuberculosis epidemic.^1^ The natural history after *Mycobacterium tuberculosis* infection has typically been categorised into either a non-infectious, asymptomatic, non-diseased state (ie, latent tuberculosis), without additional morbidity or mortality risk, or an infectious, symptomatic, diseased state (ie, clinical pulmonary tuberculosis), which is associated with increased risk of morbidity and mortality. Historically, infectious clinical tuberculosis has been used as the primary efficacy endpoint in prevention of disease (POD) tuberculosis vaccine trials, since regulators expect a reduction in clinical disease and World Health Organisation (WHO) recommends using standardised clinical endpoints.^2^^,^^3^

However, the natural history of tuberculosis is now increasingly understood to be a spectrum of states ranging from *M tuberculosis* infection through non-infectious and infectious subclinical tuberculosis to clinical tuberculosis disease.^4^ Subclinical tuberculosis is characterised by the individual not experiencing or reporting symptoms or signs of tuberculosis during screening. A review of national tuberculosis prevalence surveys suggests that subclinical tuberculosis possibly accounts for approximately 50% of all bacteriologically confirmed prevalent tuberculosis cases.^5^ Although empirical data in support of this possibility are sparse, subclinical tuberculosis might contribute to *M tuberculosis* transmission and post-tuberculosis sequelae without progression to clinical disease.^6^, ^7^, ^8^, ^9^

If subclinical tuberculosis does contribute to *M tuberculosis* transmission and tuberculosis morbidity, then understanding the efficacy of tuberculosis vaccines in preventing subclinical tuberculosis is crucial. Furthermore, tuberculosis vaccines could be more effective at preventing the more severe form of the disease and less effective at preventing subclinical disease, as has been observed in the case of COVID-19 vaccines.^10^^,^^11^

The potential health and socioeconomic effects of new tuberculosis vaccines will, therefore, depend on their efficacy in preventing clinical and subclinical tuberculosis. Newer mathematical models have considered subclinical tuberculosis when estimating the effects of tuberculosis vaccines.^12^^,^^13^ However, owing to the scarcity of empirical data, the assumptions regarding subclinical tuberculosis could be incorrect.

This Personal View summarises the potential implications of including subclinical tuberculosis in the design of POD tuberculosis vaccine trials and in the global effects of the new tuberculosis vaccines. Given that this is a developing field, we define infectious (ie, bacteriologically confirmed) subclinical tuberculosis (referred to as subclinical tuberculosis herein), in line with the recommendations from the International Consensus meeting on Early Tuberculosis held at Cape Town, South Africa, in February, 2023, as individuals with tuberculosis pathology who are capable of transmitting *M tuberculosis* infection, but are without, not aware of, or not reporting symptoms or signs of tuberculosis.^14^

## Implications of subclinical tuberculosis for POD vaccine trial design

The current standard for endpoint measurement in tuberculosis vaccine licensure trials is to monitor participants for signs and symptoms suggestive of tuberculosis and to obtain sputum for mycobacteriological investigations from participants who report symptoms. A potential disadvantage of this approach is that symptom-triggered investigations would not detect subclinical disease. There are potential benefits and limitations to including subclinical tuberculosis as a primary or secondary endpoint in tuberculosis vaccine trials.

**Figure 1:**
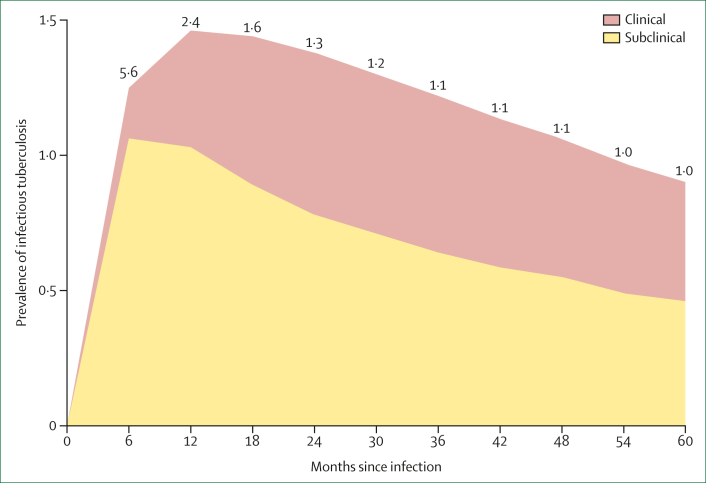
Subclinical versus clinical tuberculosis after infection Estimated relative prevalence. Numbers above the chart are the ratio of subclinical to clinical tuberculosis. Estimates from the cohort of infected individuals using *Mycobacterium tuberculosis* infection and tuberculosis disease model by Horton and colleagues.^15^

Recent modelling suggests that after *M tuberculosis* infection, the ratio of subclinical tuberculosis to clinical tuberculosis is greatest in the first 24 months (figure 1), after which, the relative proportions of subclinical tuberculosis to clinical tuberculosis are similar (figure 1).^15^ The modelling suggests that the majority of bacteriologically positive tuberculosis detected during study follow-up would be subclinical. As new infections will occur throughout the trial, the average number of months since infection in individuals with disease will be approximately half of the study period (eg, an average of 18 months for a 36-month follow-up, as in the M72 trial).^16^

An important potential disadvantage of including subclinical tuberculosis as an endpoint in POD licensure trials is that doing so could compromise the ability to show efficacy in preventing clinical tuberculosis. If active screening for subclinical tuberculosis is done during the trial, then regardless of the presence or absence of symptoms, participants diagnosed with bacteriologically confirmed subclinical tuberculosis would require treatment that would prevent the progress of subclinical tuberculosis to clinical tuberculosis, and thereby, undermine the ability to measure efficacy in preventing clinical tuberculosis, because fewer clinical cases would accrue in such a scenario.^17^ Furthermore, one-third to two-thirds of incident subclinical tuberculosis cases would be expected to regress or self-cure over the course of the trial, which could result in unnecessary treatment of such cases. If subclinical tuberculosis is included as a primary endpoint, then a substantial shift would be required in the approach undertaken by regulators.

**Figure 2:**
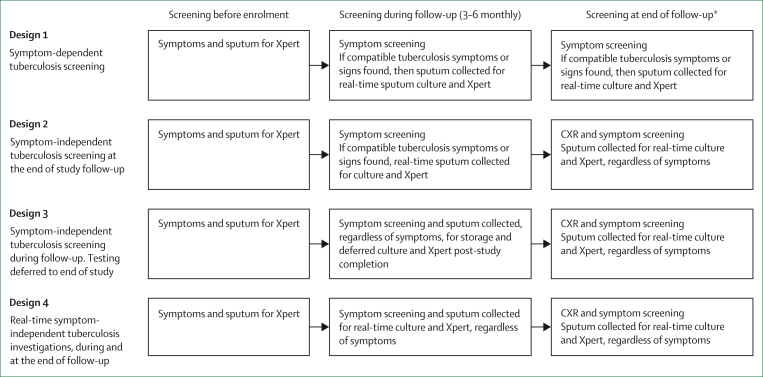
Tuberculosis screening before enrolment, during, and at the end of follow-up CXR=chest x-ray. Xpert=GeneXpert Ultra. ∗Last study visit likely to occur 2–4 years after study entry, depending on how long it takes to complete study enrolment and assuming that the last participant enrolled has at least two years of follow up.

The different design options for evaluating the efficacy of POD tuberculosis vaccines in preventing clinical and subclinical tuberculosis are shown in table 1 and figure 2, with special considerations (statistical analysis and chest radiography at study entry) described in the panel.

**Table 1:** Options for POD vaccine efficacy trial designs

	Design 1	Design 2	Design 3	Design 4∗
Analogy	M72/ASO1E IIb^14^	CORTIS,^16^ WHIP3TB,^17^ Thibela TB,^18^ H56:IC31 A055^19^	XACT,^20^ TB Fast, Track^21^	S341/A5349^22^
Phase; design	IIb/III; RCT	IIb/III; RCT	IIb/III; RCT	IIb/III; RCT
Primary objective	Efficacy in preventing cTB	Efficacy in preventing cTB	Efficacy in preventing cTB	Efficacy in preventing composite cTB-scTB
Secondary objective		Efficacy in preventing scTB	Efficacy in preventing scTB	
Ascertainment of scTB that				
Resolves	No	No	Yes	Yes
Persists	No	Yes	Yes	Yes
Progresses	No	Yes	Yes	Yes
Emerges late	No	Yes	Yes	Yes
Design implications	Does not ascertain scTB	Ascertains scTB at the end of follow-up	Ascertains scTB that emerges during study follow-up, at the end of study	Ascertains scTB real-time during study follow-up.
Sample size	+++	+++	+++	++
Study duration	+++	+++	+++	++
Cost	+++	+++	++++	++
Regulatory considerations	Accepted by regulators	May be acceptable to regulators as the design is powered to show efficacy in preventing cTB	May be acceptable to regulators as the design is powered to show efficacy in preventing cTB	Composite endpoint of scTB and cTB, not currently accepted by regulators; Could fail to show efficacy for a vaccine that prevents cTB but not scTB
Ethical considerations	scTB not detected and treated. Most will regress and those that progress to cTB during follow-up will be detected and treated. Delayed diagnosis of scTB could increase the risk of potential transmission to contacts	scTB detected and treated at the end of follow-up. scTB not detected during follow-up that progresses to cTB will be detected and treated. Delayed diagnosis of scTB may increase the risk of transmission to contacts	scTB only diagnosed at the end of follow-up. Only participants who develop cTB during follow-up would be treated, which avoids over-treatment of scTB. Delayed diagnosis of scTB may increase the risk of potential transmission to contacts	scTB detected and treated throughout follow-up, which could reduce transmission. Might over-treat scTB that would have regressed

This table defines the characteristics of the different options for POD vaccine designs.

cTB=clinical tuberculosis. POD=prevention of disease. RCT=randomised clinical trial. scTB=subclinical tuberculosis.

∗ Under the condition that regulators support using a composite primary endpoint for a POD indication.

Panel **Special considerations**

**Statistical considerations**

The analysis for the primary objectives for all designs will use Cox proportional hazards regression. Assessment of proportional hazards would be important, as including subclinical tuberculosis as a composite efficacy endpoint with clinical tuberculosis (Design 4) might mean that the proportional hazards ratio is not maintained. In Designs 2 and 3, the analysis of efficacy in preventing subclinical tuberculosis will be done separately from the analysis of efficacy in preventing clinical tuberculosis, as a secondary endpoint.

**Chest radiography**

Including a chest radiograph at study entry for Designs 2–4 might yield important information to understand the effects of the vaccine on non-infectious subclinical tuberculosis. However, implications for inclusion criteria and treatment would need to be addressed in this case. Possible options for including chest radiography at study entry include the approaches do not look, look and do not treat, and look and treat. One consideration is the number of individuals under current guidelines who would require treatment based on chest radiography findings, if both symptom and sputum Xpert screens are negative.

### Design 1

Design 1 is the typical design used for tuberculosis vaccine efficacy trials and provides no information on preventing subclinical tuberculosis. The primary objective is to evaluate efficacy in preventing bacteriologically confirmed clinical tuberculosis. At enrolment, participants are screened for symptoms and have sputum collected for Xpert, to exclude undiagnosed clinical or subclinical tuberculosis. Participants are screened at each study visit for symptoms suggestive of tuberculosis. Subclinical tuberculosis that develops during follow-up would not be detected or treated and could, therefore, transmit to contacts. Design 1 avoids detection and treatment of subclinical tuberculosis that would have resolved spontaneously. Some subclinical tuberculosis could progress to clinical tuberculosis, which would be detected by routine symptom screening. The WHO Preferred Product Characteristics for tuberculosis vaccines support this approach, and ethics committees and regulators have approved the approach for the previous and current tuberculosis vaccine efficacy trials.

### Design 2

The primary objective of Design 2 is to evaluate the efficacy of the vaccine in preventing incident bacteriologically confirmed clinical tuberculosis at the end of follow-up, and the secondary objective is to evaluate the efficacy of the vaccine in preventing subclinical tuberculosis at the end of follow-up. At entry, participants are screened for tuberculosis on the basis of symptoms and using sputum for Xpert. In addition to screening participants for symptoms suggestive of tuberculosis at every study visit, participants are screened for subclinical tuberculosis at the end of follow-up using sputum for culture or Xpert Ultra, or both, and a chest radiograph. Design 2 can only show efficacy in preventing recent or persistent (neither resolves nor progresses to clinical tuberculosis) subclinical tuberculosis. Delaying active screening for subclinical tuberculosis disease to the end of follow-up could, therefore, result in a smaller number of subclinical tuberculosis endpoints relative to the total number of subclinical tuberculosis endpoints that could accrue over the study follow-up period. Therefore, Design 2 could be under-powered to show efficacy in preventing subclinical tuberculosis but has the advantage of not recording transient subclinical tuberculosis that self-cures as an endpoint. Design 2 is modelled on the CORTIS, Thibela TB, WHIP3TB, and H56:IC31 prevention of tuberculosis recurrence (A-055) trials, which collected sputum independent of symptoms at the end of study follow-up.^18^, ^19^, ^20^, ^21^ The sample size for Design 2 would remain the same as that for Design 1, because any subclinical tuberculosis detected would not contribute to the primary endpoint. Any subclinical tuberculosis present up until the last study visit would not have been detected and treated. This approach was approved by the local ethics committees and national regulator for the CORTIS, WHIP3TB, Thibela TB, and A-055 trials, and would most likely be acceptable to ethics committees and regulatory bodies for vaccine trials, because this approach does not interfere with the standard clinical tuberculosis endpoint and allows for immediate clinical referral for all cases of detected subclinical or clinical tuberculosis.

### Design 3

Design 3 provides the most information on preventing clinical tuberculosis and subclinical tuberculosis separately. The primary objective of this design is to assess the efficacy of the vaccine in preventing bacteriologically confirmed clinical tuberculosis, and the secondary objective is to assess the efficacy of the vaccine in preventing subclinical tuberculosis. At entry, participants are screened for tuberculosis on the basis of symptoms and using sputum for Xpert. Participants are screened for symptoms suggestive of tuberculosis at each follow-up visit and investigated under the condition that symptoms are present. In addition, at each study visit, a sputum sample will be collected from the participants and frozen for culture or GeneXpert Ultra, or both, to be performed at the end of follow-up. At the end of follow-up, participants will have sputum collected for culture or Xpert Ultra, or both, and a chest radiograph. Design 3 is modelled on the Xpert Active Case-finding Trial (XACT) studies and TB Fast Track, in which specimens (sputum for culture in XACT and urine for mycobacterial culture in TB Fast Track) were collected during study but only processed at the end of follow-up.^22^^,^^23^ Design 3 will help to detect subclinical tuberculosis that later resolves or progresses to clinical tuberculosis or persists (without either resolution or progression to clinical tuberculosis) or emerges late.

Subclinical tuberculosis that resolves during study follow-up will only be detected after the end of study. Participants diagnosed with subclinical tuberculosis at the last study visit will be treated for tuberculosis. Subclinical tuberculosis that develops and progresses to clinical tuberculosis during study follow-up would be detected via symptom-triggered investigation at study visits. However, subclinical tuberculosis that could progress would not be detected early and benefit from treatment. The sample size for Design 3 would remain the same as that for Design 1, because any subclinical tuberculosis detected would not contribute to the primary endpoint. Design 3 will allow for the assessment of possible differential protective efficacy against clinical tuberculosis versus subclinical tuberculosis. Of the four designs, Design 3 is most likely to be the highest in terms of cost, owing to the collection and storage of sputum specimens at all study visits and retrospective laboratory testing at the end of study. Furthermore, ethics committees and regulators would need to approve of the collection and storage of sputum specimens during study follow-up, which would only be processed at the end of follow-up. If there is consensus that a composite clinical-subclinical tuberculosis endpoint matters as a regulatory endpoint, then the investment into a Design 3 type trial would not necessarily need to be repeated in future tuberculosis vaccine efficacy trials of the same tuberculosis vaccine candidate.

### Design 4

Design 4 uses a composite primary endpoint of clinical and subclinical tuberculosis and is likely to be the most cost-efficient of the four designs discussed herein, since the endpoints of Design 4 will accrue more rapidly, thereby allowing for a smaller sample size and shorter duration of follow-up, under the condition that Design 4 is supported by regulators and ethics committees. At entry, participants are screened for tuberculosis on the basis of symptoms and using sputum for Xpert, to exclude clinical and subclinical tuberculosis. At each study visit, participants will be screened for clinical and subclinical tuberculosis on the basis of symptoms and using sputum for culture or Xpert Ultra or both. At the end of follow-up, participants will have sputum collected for culture or Xpert Ultra, or both, and a chest radiograph. Repeated symptom-agnostic screening in Design 4 will detect bacteriologically positive subclinical tuberculosis, which would then regress; infectious subclinical tuberculosis that would persist without resolving or progressing; subclinical tuberculosis that would progress to clinical tuberculosis during study follow-up; and subclinical tuberculosis that emerges late during follow-up. Participants with subclinical tuberculosis detected during study follow-up will be referred for treatment, which would prevent progression to clinical tuberculosis, resulting in reduced numbers of clinical tuberculosis cases being detected. The net effect is that Design 4 is most likely to detect more subclinical tuberculosis than clinical tuberculosis and more primary endpoints overall than Designs 1, 2, or 3. Design 4 compromises the ability to estimate efficacy in preventing clinical tuberculosis, as detecting and treating subclinical tuberculosis is a competing risk for clinical tuberculosis. Design 4 is modelled on Study 31/A5349, which evaluated the 4-month rifapentine and moxifloxacin-based tuberculosis treatment regimen for drug-susceptible tuberculosis, in which repeated screening was done after successful completion of treatment, based on symptoms and sputum collection for Xpert and culture at each follow-up visit and at the end of study, to detect both clinical tuberculosis and infectious subclinical recurrent tuberculosis.^24^

Participants screened for subclinical tuberculosis could benefit from less diagnostic delay, improved treatment outcomes, reduced transmission, and lower costs and financial losses associated with the disease. However, the risk is that participants could be overtreated, since up to two-thirds of subclinical tuberculosis cases might regress or self-cure over the course of the trial. As Design 4 is powered using the composite endpoint and a smaller sample size, a potential risk is that a vaccine with true efficacy only against more severe phenotypes of clinical tuberculosis, but without or lower efficacy against the milder phenotype of subclinical tuberculosis, might not meet the success criterion for the primary endpoint.

Common to all proposed trial designs described herein is the risk of laboratory contamination. Although laboratory contamination could result in a false positive diagnosis of subclinical and clinical tuberculosis, in most quality-assured laboratories, contamination is rare and can be identified by strain-typing *M tuberculosis* isolates from specimens processed on the same day. The risk of including a false positive endpoint due to laboratory contamination can be reduced by requiring two *M tuberculosis-*positive cultures or Xpert screens.

## Implications of subclinical tuberculosis in the estimation of the effects of a global tuberculosis vaccine

The latest tuberculosis vaccine modelling that includes subclinical tuberculosis suggests that new POD tuberculosis vaccines could have a substantial global effect on the morbidity and mortality associated with tuberculosis.^25^^,^^26^ For example, across low-income and middle-income countries, modelling suggests that a vaccine with 50% efficacy for a 10-year duration, targeted at adolescents and adults, could prevent 44 million cases before 2050, be cost-effective or even cost-saving, increase health equity, and increase the gross domestic product by US$1·6 trillion. However, owing to the scarcity of data, this work makes assumptions on the characteristics of subclinical tuberculosis and the efficacy of new vaccines in treating subclinical tuberculosis, which could be incorrect.

At least three modelling assumptions about subclinical tuberculosis exist that, under the condition that those assumptions are incorrect, could lead to higher model-estimated effects of the vaccine on the global tuberculosis burden (table 2). First, the current model assumes that the vaccine is not protective when given to individuals with prevalent subclinical tuberculosis at the time of vaccination. If instead, the vaccine is protective when given to individuals with subclinical tuberculosis, then the model-estimated effect would be higher. Second, current modelling assumes that the vaccine-induced protection is lost when individuals progress from *M tuberculosis* infection to subclinical tuberculosis. If instead, the protection is retained when individuals progress from *M tuberculosis* infection to subclinical tuberculosis, then the model-estimated effect would be higher. Finally, the current model assumes no morbidity from subclinical tuberculosis. If instead, subclinical tuberculosis results in some morbidity, which is most likely, given the pathological damage that is needed for *M tuberculosis* to be detected in respiratory samples, then the model-estimated effect would be higher.^27^^,^^28^

**Table 2:** Implications of wrong key assumptions about subclinical tuberculosis in the current model on the model-estimated impact

	The most likely effect of the assumption being wrong on the model-estimated health and economic impact of the vaccine
Vaccine not effective in individuals with subclinical tuberculosis	Higher global impact
Vaccine efficacy lost upon progression from *Mycobacterium tuberculosis* infection to subclinical tuberculosis	Higher global impact
No morbidity from subclinical tuberculosis	Higher global impact
Subclinical tuberculosis slightly less infectious than clinical tuberculosis	Higher or lower global impact
Vaccine efficacy reduces upon progression from *M tuberculosis* infection to subclinical tuberculosis	Lower global impact
No self-resolution of subclinical tuberculosis to *M tuberculosis* infection	Lower global impact

At least one subclinical tuberculosis-related assumption exists that, under the condition that the assumption is incorrect, could lead to higher or lower model-estimated effects of the vaccine on the global tuberculosis burden. The current modelling studies assume the infectiousness of subclinical tuberculosis to be 83% that of active tuberculosis.^25^^,^^26^ If the infectiousness of subclinical tuberculosis is not 83% that of active tuberculosis (either because subclinical tuberculosis is more or less infectious, or of shorter or longer duration), then the model-estimated effect of the vaccine would be higher or lower. Given the wide range of assumed infectiousness in recent analyses, the effect on estimates should be considered.^9^^,^^29^

At least two subclinical tuberculosis-related assumptions exist that, under the condition that those assumptions are incorrect, could lead to lower model estimates of the effect of the vaccine on the global tuberculosis burden. First, the current modelling^25^^,^^26^ assumes that vaccination does reduce the probability of individuals progressing from *M tuberculosis* infection to subclinical tuberculosis. If instead, previous vaccination does not reduce the probability of individuals progressing from *M tuberculosis* infection to subclinical tuberculosis, then the model-estimated effect of the vaccine would be lower. Finally, the current model assumes that subclinical tuberculosis does not resolve spontaneously to *M tuberculosis* infection. If instead, subclinical tuberculosis did resolve spontaneously to *M tuberculosis* infection, as suggested by a recent review and analysis of prechemotherapy data, then the model-estimated effect of the vaccine would be lower.^7^^,^^30^

## Conclusions

Subclinical tuberculosis accounts for almost half of all tuberculosis in national tuberculosis prevalence surveys and could be associated with morbidity, in addition to contributing substantially to transmission. Thus, evaluating the efficacy of POD tuberculosis vaccines is pertinent to preventing clinical and subclinical tuberculosis. Several design options with various benefits, limitations, and ethical considerations are considered that would allow us to generate the evidence needed to estimate the effect of the vaccines on the global tuberculosis burden, and to inform policy and vaccination strategies. Regulatory authorities would need to be willing to include subclinical tuberculosis as an efficacy endpoint.

## Declaration of interests

The following authors declare grants to their respective institutions. GJC: Division of AIDS/NIH. ALF-G: Bill & Melinda Gates Foundation, US NIH for HIV Vaccine Trials Network Statistical Center. ALG-B: Barcelona Institute for Global Health tenure track associate research professorship. EBW: Burroughs Wellcome Fund, 1022002; Bill & Melinda Gates Foundation, INV-046520; NIH/National Institute of Allergy and Infectious Diseases, AI201700104 and AI147321. FC: Bill & Melinda Gates Foundation, EDCTP. HE: Canadian Institutes for Health Research, Tier 1 Canada Research Chair. VM: NIH, Centers for Disease Control and Prevention. MH: NIH, EDCTP, Gates Medical Research Institute. RMGJH: European Research Council. RGW: Wellcome Trust (218261/Z/19/Z), NIH (1R01AI147321-01, G-202303-69963), EDCTP (RIA208D-2505B), UK Medical Research Council (CCF17-7779 Bloomsbury), Economic and Social Research Council (ES/P008011/1), Bill & Melinda Gates Foundation (INV-004737, INV-035506), WHO (2020/985800-0). ADG, WAH, MB, ACS, KF, AFD, and MXR declare no competing interests.

The following authors declare royalties. ALG-B: Royalties for the patent Molecular differences between species of the *Mycobacterium tuberculosis* complex in November, 2020 and November, 2021. HE: Royalties to Stanford University for the patent Molecular differences between species of the *Mycobacterium tuberculosis* complex in November, 2020 and November, 2021.
